# Intravital Microscopy of Lipopolysaccharide-Induced Inflammatory Changes in Different Organ Systems—A Scoping Review

**DOI:** 10.3390/ijms242216345

**Published:** 2023-11-15

**Authors:** Cassidy Scott, Daniel Neira Agonh, Hannah White, Saki Sultana, Christian Lehmann

**Affiliations:** 1 Department of Anesthesia, Pain Management and Perioperative Medicine, Dalhousie University, Halifax, NS B3H1X5, Canada; cassidy.scott@dal.ca; 2 Department of Pharmacology, Dalhousie University, Halifax, NS B3H1X5, Canada; hn293510@dal.ca (H.W.); saki.sultana@dal.ca (S.S.); 3 Department of Physiology and Biophysics, Dalhousie University, Halifax, NS B3H1X5, Canada; d.neira@dal.ca

**Keywords:** intravital imaging, lipopolysaccharide, leukocytes, microcirculation, inflammation

## Abstract

Intravital microscopy (IVM) is a powerful imaging tool that captures biological processes in real-time. IVM facilitates the observation of complex cellular interactions in vivo, where ex vivo and in vitro experiments lack the physiological environment. IVM has been used in a multitude of studies under healthy and pathological conditions in different organ systems. IVM has become essential in the characterization of the immune response through visualization of leukocyte–endothelial interactions and subsequent changes within the microcirculation. Lipopolysaccharide (LPS), a common inflammatory trigger, has been used to induce inflammatory changes in various studies utilizing IVM. In this review, we provide an overview of IVM imaging of LPS-induced inflammation in different models, such as the brain, intestines, bladder, and lungs.

## 1. Introduction

The unique advantage of intravital microscopy (IVM) is its capability of capturing biological processes in real-time. IVM facilitates the observation of cellular interactions in vivo in the physiological environment. IVM has been used for studies under healthy and pathological conditions in different organ systems. In particular, IVM has become utilized in the characterization of the immune response through visualization of leukocyte–endothelial interactions and subsequent changes in the microvascular perfusion. Lipopolysaccharide (LPS), a common inflammatory trigger, has been used to induce inflammatory changes in various studies utilizing IVM. In this review, we provide an overview of IVM imaging of LPS-induced inflammation in different models, such as the brain, intestines, bladder, and lungs. The scoping review was performed by searching the PubMed database using the following keywords: microcirculation, live imaging, inflammation, IVM, LPS, and leukocyte interactions.

Intravital imaging of biological processes requires specific microscopy techniques, such as fluorescence microscopy. Transillumination is a common method of fluorescence microscopy that uses the light of specific wave lengths to excite fluorescence dyes within biological samples [1]. A barrier filter absorbs the light used for illumination while permitting fluorescence transmission and allowing for the visualization of a bright object over a dark background. One drawback to this method is that thick and dense tissues cannot be visualized [1,2]. Another method of fluorescence microscopy is epi-illumination. In this technique, light goes through the objective, concentrating the light source, and thereby illuminating the object [2]. Emission light is then collected through the objective. This technique builds on the weaknesses of transillumination, allowing for the visualization of thicker tissues [2].

In addition to differences in fluorescence techniques, there are also different optical methods whose uses are dependent on visualization targets and resources: wide-field, confocal, multiphoton, and spinning disk microscopy. Wide-field microscopy is a cost-effective means to analyze tissues when deep tissue penetration is not required [3]. This technique excites fluorochromes by using a beam of light to illuminate the field of view of the specimen. Confocal microscopy improves on wide-field microscopy by replacing the beam of light with a pinhole, decreasing the amount of out-of-focus light, and improving image resolution [3]. This method is relatively cost-effective when compared to other techniques but has a limited depth of penetration [4]. In addition, the time required for imaging is long, which leads to an increased risk of phototoxicity [2]. Multiphoton microscopy allows for better control by using lasers to excite fluorescence, as the fluorophore requires two photons for excitation [4]. This technique produces less phototoxicity and photobleaching and has an increased depth of penetration [2,4]. Lastly, spinning disk microscopy improves confocal microscopy by adding additional pinholes, thereby increasing image acquisition speed and reducing phototoxicity [2].

Usually, exogenous fluorochromes, such as fluorescein isothiocyanate (FITC) and rhodamine-6G, are administered intravenously to experimental animals [5]. FITC bound to albumin is used to visualize blood vessels, including arterioles, capillaries, and venules, and to study capillary leakage. When excited with wavelengths between 450 and 490 nm, fluorescence within or outside the vessels can be quantified [2]. Rhodamine-6G is used to label leukocytes by selectively binding to mitochondria. Red blood cells lack mitochondria, allowing leukocytes to be visualized when fluorochromes are excited with light in the 515–560 nm range [5]. Using a combination of both fluorochromes allows for studies of leukocyte–endothelial interactions and microvascular perfusion following an inflammatory challenge [6,7,8].

LPS is found in the outer membrane of Gram-negative bacteria [9]. It is composed of three structural domains: Lipid A, a core oligosaccharide, and the O-antigen. LPS acts as a pathogen-associated molecular pattern (PAMP) to activate specific pattern recognition receptors in cells. Specifically, LPS activates Toll-like receptors (TLR) 2 and 4, inducing proinflammatory changes in cells [10,11]. For activation, LPS-binding protein (LBP), CD14, and MD-2 are involved to extract and shuttle LPS to the receptor [12,13]. Firstly, LBP targets LPS in Gram-negative bacteria, solubilizing the molecule. Next, the LPS is transferred to CD14, which exists on the plasma membrane of the cell or in a soluble form. Once bound, the LPS molecule binds with MD-2 associated with TLR, causing a conformational change of the receptor and promoting homodimerization with a second TLR [14].

Activation of the receptors promotes TRIF and MyD88 intracellular signaling cascades, ultimately resulting in the expression of cytokines, cellular adhesion molecules, and other pro-inflammatory mediators by the NF-kB, AP-1, and IRF-3 pathways. LPS is commonly administered as a method for generating sepsis in an infection-free animal model [15]. Common routes of LPS administration for initiating inflammatory changes in animal models are intravenous or intraperitoneal. Using IVM, LPS-induced leukocyte–endothelial interactions can be visualized [15,16]. Mucins and integrins aid in leukocyte slowing, rolling, adhesion, and ultimately extravasation to the site of infection. With IVM, video acquisition allows for analysis of these immune interactions, among other specific parameters.

Following image acquisition, analysis of IVM videos occurs offline. ImageJ 1.45 (accessed on 8 July 2023) is a commonly used software for the quantification of leukocyte rolling and adhesion [17]. Commonly assessed outcomes for IVM include number of adherent and/or rolling leukocytes, leukocyte rolling flux/flow, and functional capillary density (FCD). Adherent leukocytes are typically defined as adherent to the endothelia for 30 s and expressed as the number of adherent leukocytes per mm^2^. Rolling flux or flow is commonly defined as the number of leukocytes passing a vessel diameter observed per minute, although variability has been seen in the definition of leukocyte rolling. Some researchers define rolling leukocytes as those that interacted with but did not adhere to the endothelia over a specific distance [18], while others define rolling leukocytes as those moving at a velocity less than that of erythrocytes [19]. Lastly, functional capillary density (FCD) is commonly defined as the length of capillaries with observable erythrocyte perfusion.

## 2. Intravital Imaging of the Brain

Sepsis-associated encephalopathy (SAE) is a model commonly used to assess the microvasculature of the central nervous system (CNS) by IVM. LPS used to induce sepsis damages the function of the blood–brain barrier (BBB) and promotes the development of SAE. The BBB acts as a shield, protecting the CNS from toxic substances in the circulatory system by controlling the exchange between the blood and brain tissue [20]. In healthy subjects, the BBB has low permeability, mainly determined by tight junctions and adherens junctions formed by intercellular cadherins and intracellular catenin. However, several studies have demonstrated the ability of LPS to enhance BBB permeability by disrupting tight junctions and adherens junctions, allowing toxic substances to cross the BBB and induce CNS dysfunction [21]. When the barrier function is damaged, immune cells can penetrate brain tissue, causing a local inflammatory response. Studies by Hailesalassie et al. (2020) have shown that LPS administration in mice causes an increase in vascular leukocyte adhesion factors, including vascular cell adhesion molecule-1 (VCAM-1) and intercellular adhesion moleculue-1 (ICAM-1), which are involved in a series of cellular responses such as leukocyte recruitment and migration [22]. IVM allows us to visualize these microcirculatory alterations in vivo following LPS administration.

### 2.1. Methods

IVM of the brain requires the installation of a cranial window for direct observation of the cerebral microcirculation. There are two main methods for creating cranial windows: (1) transcranial windows created by thinning the cranium, and (2) implanted windows that replace a region of the cranium. However, implanted windows, such as glass coverslip-based cranial windows, are most commonly used in IVM procedures. In order to create a cranial window, the animal is first anesthetized and placed on a stereotaxic frame. The skin and muscle are then removed, and a window, typically varying between 2 and 5 mm in diameter, is drilled into the skull to access the brain parenchyma. A coverslip is then placed over the exposed brain, and animals are left for a determined number of days to recover before IVM is scheduled. This process requires sterile techniques to avoid local infections. The method has been used for imaging access for up to 175 days [23]. The cranial vault must not be opened to ensure physiological pressures and blood flow are maintained [24]. Following the implantation process, IVM can be performed through the administration of fluorescent dyes. Although rhodamine 6G and FITC-albumin are some of the most common fluorescents used in intravital imaging of the brain, others can be used. Ruiz-Valdepeñas et al. (2011) administered 70,000 MW Texas red-conjugated dextrane solution to stain the intravascular space while leaving nucleated cells unstained. Using this method, only cells that are stationary or dramatically slowed by adhesive interactions can be detected, allowing researchers to study leukocyte margination [25]. Under normal conditions, this solution is unable to leave the vascular bed, but when the integrity of the BBB is compromised, such as following LPS administration, extravasation of the fluorescently labeled dextrane occurs [25]. Additionally, genetically encoded fluorescent proteins (FPs) are also commonly used for in vivo imaging and allow for cell tracking during development. Kim et al. (2020) used LysMGFP/+ and CX3CR1GFP/+ mice for analysis of the brain microcirculation, in which the lysozyme and CX3CR1 genes are replaced by green fluorescent protein (GFP), respectively. LysMGFP/+ mice were then administered 70,000 MW Texas red dextrane for visualization of the blood vessels. CX3CR1GFP/+ were administered a PE-conjugated anti-Ly6G antibody for observing neutrophils [26]. However, Far-Red fluorescent proteins (RFPs) are preferred over GFPs due to lower light absorption by hemoglobin, minimizing autofluorescence [2]. Lastly, fluorescently labeled antibodies against specific cell receptors have been used for intravital imaging of the brain. Adhesion molecules expressed during migration, such as ICAM-1, VCAM-1, and PECAM-1, can be targeted, by antibodies to study vascular cell migration. For example, Jenne et al. (2011) used rat anti-mouse PECAM-1 mAb conjugated to Alexa Fluor 488 to visualize recruitment in the mouse brain microvasculature [24].

### 2.2. Results

**Figure 1:**
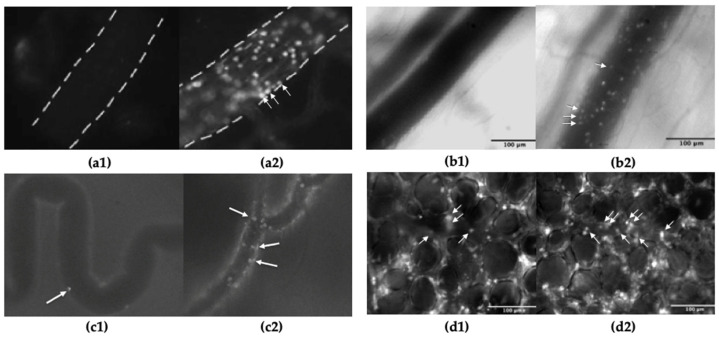
Image captures of leukocyte adhesion in control vs. LPS-stimulated animals, visualized by intravital microscopy in different organ systems; (**a1**) brain IVM from a control vs. (**a2**) LPS-stimulated animal, adapted from Zhou et al. [33]; (**b1**) intestinal IVM from a control vs. (**b2**) LPS-stimulated animal, adapted from Hall et al. [34]; (**c1**) bladder IVM from vs. (**c2**) LPS-stimulated animal, adapted from Hagn et al. [6]; (**d1**) lung IVM from a control vs. (**d2**) LPS-stimulated animal, adapted from Hall et al. [34]; arrows indicate adherent leukocytes.

Results of these studies have consistently found LPS administration to increase leukocyte recruitment into the brain microcirculation across varying dosages (Table 1) (Figure 1). Ruiz-Valdepeñas et al. (2011) performed IVM using a cranial window to study sepsis triggered by an intravenous (i.v.) injection of LPS. The results from this study found a significant increase in the density of marginated leukocytes in LPS-treated animals compared to vehicle-treated animals, where leukocyte margination was not seen at any timepoint. Additionally, LPS significantly increased the extent of dextrane extravasation out of the blood vessel, indicating compromised integrity of the BBB [25]. Similar results were found by Wu et al. (2016), who found that LPS administration significantly increased leukocyte rolling flux and leukocyte adhesion in postcapillary venules of the CNS [19]. These results were later confirmed in a second study conducted by the same research group, finding that LPS administration significantly increased leukocyte rolling flux and leukocyte adhesion compared to saline control [27]. Varying timepoints have been used to assess the inflammatory response to LPS administration. Ramirez et al. (2010) found that administration of LPS significantly increased leukocyte adhesion from baseline at 4 and 24 h. At four hours, leukocyte adhesion was increased by ≈50-fold, which decreased to a ≈35-fold increase by twenty-four h [28]. Similar results were seen in a second study by the same group. They found that LPS administration increased leukocyte adhesion from baseline at 4 h by ≈30-fold, which decreased to an ≈18-fold increase by 24 h compared to baseline [29]. Wang et al. (2019) also assessed brain microcirculation at varying timepoints (0, 1, 2, 4, and 24 h) following LPS administration. They found that 2 h following LPS instillation leukocyte adherence to microvessels was significant and did not recover by the 24 h timepoint. Additionally, LPS instillation significantly increased vascular permeability, as assessed by FITC-albumin fluorescence leakage into the perivascular areas [30]. Studies by Zhou et al. (2012) examined an acute 2 h timepoint and found that LPS administration significantly increased leukocyte adhesion in the pial vessels compared to control animals. Furthermore, LPS significantly decreased FCD compared to control [18]. Lastly, studies by Li et al. (2023) examined the effects of acute lung injury (ALI) on the BBB. They found that three doses of intranasal (i.n.) LPS (10 μg/40 μL) induced neutrophil adhesion to the cerebral endothelium in the BBB and neutrophil extravasation from the cerebral vasculature [31].

The majority of studies examine leukocyte extravasation in male mice. However, Hughes et al. (2013) examined the influence of sex on LPS-induced leukocyte–endothelial cell interactions. The administration of LPS significantly increased leukocyte adherence, emigration, and rolling velocity in the cerebral vascular bed of both sexes compared to the control animals. However, the observed LPS-induced increase in leukocyte rolling velocity was significantly increased in LPS-challenged females compared to males [32]. Future studies should examine potential sex-dependent differences in LPS-induced microvasculature dysfunction.

**Table 1:** Impact of LPS administration on IVM readouts in the brain microcirculation.

Animal Strain	Dosage of LPS	Readout	Result	References
C57BL/6J mice	1 mg/kg, i.v.	Neutrophil and platelet recruitment in postcapillary venules.	LPS induced neutrophil and platelet recruitment in the brain. Both cell types were seen adhering to the vascular endothelium.	[24]
C57BL/6J mice, male and female	10 μg/mouse, i.p.	Leukocyte adherence, emigration, and rolling velocity in the cerebral vascular bed.	LPS significantly increased leukocyte adherence and rolling velocity.	[32]
C57BL/6J mice, male	7.5 mg/kg/h over two hours, i.v.	Leukocyte adherence and vascular permeability in cerebral venules at 0, 1, 2, 4, and 24 h.	LPS significantly increased leukocyte adherence by the 2 h timepoint. which did not recover by the 24 h timepoint. Additionally, LPS significantly increased vascular permeability.	[30]
C57BL/6J mice, male	2 μL of 2 μg LPS, intracerebroventricular injection	Leukocyte rolling flux and adhesion in postcapillary venules.	LPS significantly increased leukocyte rolling flux and adhesion compared to saline control.	[19]
C57BL/6J mice, male	2 μL of 2 μg LPS, intracerebroventricular injection	Leukocyte rolling flux and adhesion in postcapillary venules.	LPS significantly increased leukocyte rolling flux and adhesion compared to saline control.	[27]
C57BL/6J mice, male	LPS 6 mg/kg, i.p.	Leukocyte adhesion in brain endothelium at 0, 4, and 24 h.	LPS increased leukocyte adhesion from baseline by ≈50-fold at 4 h, decreasing to a ≈35-fold increase by 24 h.	[28]
C57BL/6J mice, male	LPS 6 mg/kg, i.p.	Leukocyte adhesion in brain endothelium at 0, 4, and 24 h.	LPS increased leukocyte adhesion from baseline by ≈30-fold increase, decreasing to an ≈18-fold increase by 24 h.	[29]
Lewis rats, male	LPS 5 mg/kg, i.v.	Leukocyte adhesion, roller flow in pial vessels, and functional capillary density (FCD).	LPS administration significantly increased leukocyte adhesion. Furthermore, LPS significantly decreased roller flow and FCD.	[18]
C57BL/6J mice	LPS 1 mg/kg, i.v.	Leukocyte margination in subpial vessels.	LPS significantly increased the density of marginated leukocytes.	[25]
C57BL/6J mice	Three doses of LPS 10 μg/40 μL, i.n.	Neutrophil and blood–brain barrier permeability.	LPS signficantly increased neutrophil adhesion and extravastion into the parenchyma.	[31]

## 3. Intravital Imagining of the Intestines

### 3.1. Methods

The use of IVM for studying LPS-induced microcirculatory changes in the gastrointestinal tract is relatively common compared to investigating microcirculation by IVM in other organs. Due to the unique microvascular anatomy of the intestine, which makes the intestine particularly vulnerable to microcirculatory disturbances due to inflammation, the intestine is a choice of organ for studying microcirculatory changes in different chronic and acute inflammations. The available literature has reported different doses and routes for LPS administration to induce chronic and acute inflammations for studying intestinal microcirculatory changes in murine. The IVM process requires exteriorizing a loop of the terminal ileum from the abdominal cavity. The exposed intestines are bathed in a continuous flow of warm saline to mimic physiological conditions. Some researchers administer isoproterenol in a drop-wise manner to reduce peristaltic movement [35].

In addition to parameters that are commonly measured by IVM (i.e., leukocyte–endothelial interactions and FCD), intestinal permeability, venular wall shear rate, circulating platelets, etc. are also commonly measured by intestinal IVM. In most of the reported studies, acute inflammation caused by LPS administration resulted in increased leukocyte adhesion and decreased FCD in the intestinal microcirculation (Figure 1) (Table 2) [36,37].

### 3.2. Results

For studying intestinal microcirculation by IVM, fluorescence microscopy is the most used tool where blood cells and other components of circulatory systems are fluorescently labeled. Most studies have used the epifluorescence modality due to its ease of use. Transillumination modality has been reported in very few studies. Schmidt et al. reported one such study where transillumination fluorescence microscopy was performed during IVM to determine microvascular permeability and leukocyte adhesion in male rats [38]. Intravenous infusion of LPS at a dose of 4 mg/kg/h was used in this study to induce endotoxemia in Wistar rats. Transillumination modality was used in this study, finding that treatment with LPS significantly increased microvascular permeability and leukocyte adhesion and decreased venular wall shear rate. The dosage of LPS used in this study was identified in pilot experiments, finding that it would not cause pronounced hypotension and a significant inflammatory endothelial compared to nonendotoxemic animals.

Perry et al. also used transillumination microscopy for studying intestinal microcirculation in rats [39]. But instead of using conventional LPS of *E. coli* origin, this group used bacterial endotoxin originating from *S. abortus* equi. In this study, they examined leukocyte adherence at different layers of the intestine and observed that adherent or rolling leukocytes did not change in the mucosa upon administration of LPS. However, in the submucosa and muscularis, they noted a low level of adherent leukocytes and greatly increased rolling leukocytes after the LPS challenge. This differential leukocyte adherence across the intestinal wall was explained by the gradient expression of ICAM-1 and P-selectin across the gut wall. These adhesion molecules are expressed at a very low level at the mucosa, with a gradual increase in expression through the submucosa and muscularis. Interestingly, this study reported elevated expression of P-selectin upon endotoxin treatment, whereas no changes in the expression of ICAM were reported. However, some previous studies reported the upregulation of ICAM expression in rat intestines upon LPS challenge [37,38]. *S. abortus* equi-originated endotoxin could be a reason for this discrepancy reported in their finding. The differences in the basal expression of ICAM could be due to the different sources of endotoxin used in their study or could be due to the differences in animal strains being used in those studies.

Mangell et al. investigated the role of P-selectin-dependent leukocyte recruitment in LPS-induced intestinal barrier dysfunction in mice [40]. This study is particularly important as it showed that LPS-induced leukocyte recruitment is mediated by P-selectin. The study also emphasized that sepsis-associated intestinal leakage in the gut is largely regulated by leukocyte accumulation. This is a noble finding that establishes a critical link between P-selectin-dependent leukocyte recruitment and gut barrier failure in endotoxemia. The host response to the LPS challenge is associated with disturbed gut integrity and increased leukocyte infiltration. Increased intestinal leakage through the epithelial cell lining is a key feature in the pathophysiology of sepsis, as it facilitates the bacterial translocation and passage of toxic substances from the gastrointestinal tract.

Kamp et al. reported a study where they assessed neutrophil recruitment in the intestinal microcirculation during acute inflammation [41]. This group performed IVM of the small intestine (jejunum) of mice after 1 h and 4 h of the LPS challenge. Interestingly, after 1 h, the number of rolling and adherent neutrophils did not change in LPS-challenged mice compared to saline-induced control animals. However, after 4 h of LPS exposure, they observed an elevation of both parameters in the intestinal microcirculation. The amplified immune responses that occurred at later time points could be due to delayed immune responses to the subsequent increased bacterial load in the intestinal tissue. This study emphasized the fact that initial rolling and tethering may change over time. The adhesion molecules P- and L-selectins contribute to initial tethering but start shedding off after a certain period. The entire dynamics of tethering and shedding of selectins are important at any time point. So, the experimental models and time points chosen for the IVM should be carefully considered while interpreting the experimental outcomes.

Although LPS administration can induce sepsis pathophysiology in experimental animals and trigger various inflammatory pathways in vivo, it does not mimic the clinical course of the condition; it can only generate hyperinflammation [35]. Future research should use a more clinically relevant model of sepsis, where an infection is present. The available literature suggests that longer endotoxemia models would be more useful to observe clinically relevant intestinal microcirculation changes caused by systemic inflammation.

**Table 2:** Impact of LPS administration on IVM readouts in the intestinal microcirculation.

Animal Strain	Dosage of LPS	Readout	Result	References
Male Wistar rats	i.v. infusion of LPS (*E. coli*), (5 mg/mL stock in saline) administered via the jugular vein at 4 mg/kg/h rate	Leukocyte adhesion, vascular leakage and venular wall shear rate.	LPS increased leukocyte adhesion. LPS infusion caused a significant increase in macromolecular leakage, decreased venular wall shear rate	[38]
Rats	LPS (*E. coli*) (5 mg/kg); i.v.	Leukocyte adhesion and FCD in the muscle layer and mucosa	LPS increased leukocyte adhesion. LPS decreased FCD in longitudinal muscle layer, circular muscle layer, and mucosa.	[36]
Lewis rats	LPS (5 mg/kg), i.v.	Leukocyte adhesion and FCD in the muscle layer and mucosa	LPS increased leukocyte adhesion. LPS decreased FCD in the longitudinal muscle layer, circular muscle layer, and mucosa. LPS increased non-functional capillaries.	[37]
Transgenic mice with 129/SvJ background	LPS (*E. coli*) (0.5 mg/kg); i.p.	Leukocyte adherence, rolling, and epithelial barrier permeability.	LPS increased adhesion but decreased number of rolling leukocytes and increased neutrophil infiltration into the intestinal tissue. LPS increased epithelial barrier permeability.	[42]
C57BL/6J mice	LPS (*E. coli*) (0.5 mg/kg); i.p.	Leukocyte rolling and adhesion.	LPS increased rolling neutrophils and adherent neutrophils 4 h after administration.	[41]
C57BL/6 mice	LPS (*E. coli*), (20 mg/kg); i.p.	Leukocyte rolling and adhesion. Intestinal permeability, vessel diameter, and blood flow.	LPS increased leukocyte adhesion and rolling. LPS increased intestinal permeability and vessel diameter. LPS decreased blood flow.	[40]
Sprague ± Dawley rats	LPS *(E. coli)*, 15 mg/kg via jugular vein	Leukocyte rolling velocity and adhesion.	LPS increased adhesion and migration of leukocytes. LPS decreased rolling velocity.	[43]
Lewis rats	LPS (*E. coli*) (5 mg/kg); i.v.	Leukocyte adhesion and rolling. FCD in the muscle layer and mucosa.	LPS increased leukocyte adhesion and decreased rolling. LPS decreased FCD with an increase in number of dysfunctional capillaries in muscularis longitudinalis/circularis, and mucosa.	[44]
C57BL/6 mice	LPS (*E. coli* or *K. pneumoniae*), (5 mg/kg); i.v.	Leukocyte adhesion and FCD in the muscle layer and mucosa.	LPS increased leukocyte adhesion and decreased FCD.	[35]
Wistar Kyoto rats	LPS (*E. coli)*, (2 mg/kg); i.v.	FCD of the muscle layer	LPS had no significant difference in FCD.	[45]
Sprague-Dawley rats	endotoxin (*S. abortus equi)*, (0.5–2.0 mg/kg);	Leukocyte rolling and adhesion in the mucosa, submuscosa, and muscularis.	LPS had no effect on adhesion or rolling of leukocytes in the mucosa. Low-level adhesion and significantly increased rolling leukocytes were found in submucosa and muscularis.	[39]
Sprague-Dawley rats	LPS (*E. coli*), (10 mg/kg); i.v.	Leukocyte rolling and adhesion. Circulating platelets.	LPS increased rolling and firm adhesion of leukocytes. LPS decreased circulating platelets.	[46]

## 4. Intravital Imaging of the Bladder

### 4.1. Methods

Although the use of IVM to study the bladder microcirculation is well established, there are only a few IVM studies that specifically use LPS to induce inflammatory changes in the bladder microcirculation. The three studies examined in this review used a murine model for IVM, which was adapted from a previously used rat model by Kowalewska et al. in 2011 [47]. For bladder IVM, the urinary bladder must be exposed, and the bladder is filled with saline administered via a catheter. Female animals are typically used, as male animals have a longer urethra that makes transurethral catheterization quite difficult [48]. The bladder is exteriorized as much as possible and covered with a glass coverslip for imaging.

The administration of LPS occurs prior to imaging, but the time between LPS administration and imaging varied slightly between groups. Kowalewska et al. began imaging 4 h post-LPS administration [47], while both Hagn et al. and Berger et al. began their imaging only 2 h post-LPS administration [6,49]. Despite these differences, these timepoints are all considered acute models of inflammation.

The studies reviewed also varied in their imaging techniques. Kowalewska et al. used an inverted intravital microscope and transillumination to visualize the microcirculation. They positioned the mouse in a lateral position and fully exteriorized the bladder to allow for transillumination in the inverted microscope. On the other hand, both Hagn et al. and Berger et al. used a widefield epifluorescence microscope with rhodamine-6G to visualize the leukocytes and FITC-albumin to visualize the capillary beds. Their mice were in a supine position for the duration of the imaging. However, the variation in visualization techniques between groups is unlikely to impact the results. A study comparing transillumination to FITC-dextran and rhodamine for visualization of the intestinal microcirculation found that there were no significant differences in leukocyte adherence and rolling between groups [50].

### 4.2. Results

The administration of LPS was found to increase leukocyte adhesion in all studies reviewed, no matter the method of administration (Figure 1) (Table 3). Berger et al. found intravesical LPS administration to be superior at increasing adhesion when compared to intraperitoneal LPS administration [49]. The other studies examined exclusively used intravesical administration for LPS [6,47]. This is consistent with work comparing intravesical and intraperitoneal LPS administration in rats. It was found that intravesical LPS administration produced a greater increase in inducible nitric oxide synthase, used as a marker for inflammation, when compared to intraperitoneal LPS administration [51].

Different concentrations of LPS seemed to cause varying amounts of leukocyte adhesion. Kowalewska et al. did not observe a significant increase from baseline leukocyte adhesion with LPS administration at a dose of 0.1 mg/kg or 1.0 mg/kg. They only saw significant changes with a dose of 5.0 mg/kg, four hours after administration. However, both Berger et al. and Hagn et al. observed a significant increase in leukocyte adhesion with a dose of 0.375 mg/kg two hours after administration. These differences could be due to variations in LPS administration. Kowalewska et al. held the LPS in the bladder for 15 min to induce inflammation, while both Berger et al. and Hagn et al. held the LPS in the bladder for 30 min.

The differences in leukocyte adhesion observed could also be partially accounted for by the bacterial sources of LPS used. Kowalewska et al. compared LPS from *E. coli* and *P. aeruginosa*. They found that LPS from *E. coli* increased leukocyte adhesion sooner and at a lower dose when compared to LPS from *P. aeruginosa*. Even among results using LPS from the same bacterial species, adhesion differed with different serotypes. The LPS used by Kowalewska et al. was *E. coli* serotype 0127:B8, and they observed increased adhesion at 5.0 mg/kg after four hours. However, the studies by Berger et al. and Hagn et al. used LPS from *E. coli* serotype 026:B6 and observed increased adhesion at 0.375 mg/kg after two hours. Indeed, *E. coli* serotypes with varying O antigen structures have been reported to activate the immune response differently, even if they all stimulated an inflammatory response. For example, differing serotypes of LPS isolated from *E. coli* were found to activate different inflammatory transcription factors and produce differing levels of inflammation in a murine model of preterm birth [52].

Another factor that could account for some of the differences in leukocyte adhesion observed is the mouse strain used, as immune responses can differ based on genetic background. Kowalewska et al. used C57BL/6 mice, while both Berger et al. and Hagn et al. used BALB/c mice. C57BL/6 mice tend toward a TH1-type immune response, while BALB/c mice produce a TH2-type immune response [53]. This difference manifests itself in many aspects of the immune response. One study of interstitial cystitis found that BALB/c mice had a significantly increased mast cell count in the bladder in response to pseudorabies virus infection when compared to C57BL/6 mice [54]. Another study using a murine model of sepsis found that levels of leukocyte infiltration into tissue and inflammatory cytokines were significantly increased in BALB/c mice when compared to C57BL/6 mice [55]. Since the studies using BALB/c mice observed significant changes in leukocyte adhesion at a lower concentration of LPS than those using C57BL/6 mice, it is possible that this is in part due to a more robust immune response from the BALB/c strain.

LPS administration was found to decrease functional capillary density (FCD) in those studies that examined it as an outcome. Hagn et al. did not find a statistically significant decrease; however, Berger et al. found significance with both intraperitoneal and intravesical LPS administration. Interestingly, both groups used the same method for intravesical LPS administration: 150 μg/mL of LPS in 50 μL of saline held in the bladder for 30 min. They also both used FITC-albumin for visualization of the capillary beds, and both began imaging two hours after LPS administration [6,49].

**Table 3:** Impact of LPS administration on IVM readouts in the bladder microcirculation.

Animal Strain	Dosage of LPS	Readout	Results	References
Female CD-1 (i.p.) Female BALB/c (intravesical)	20 mg/kg i.p. 0.375 mg/kg intravesical held for 30 min	Leukocyte adhesion and functional capillary density	i.p. and intravesical LPS significantly increased adhesion and decreased FCD.	[49]
Female BALB/c	0.375 mg/kg intravesical held for 30 min	Leukocyte adhesion and functional capillary density	LPS significantly increased adhesion. LPS decreased FCD, but not significantly.	[6]
Female C57BL/6	0.1, 1.0, 5.0, or 7.0 mg/kg intravesical held for 15 min	Leukocyte adhesion and rolling	5 and 7 mg/kg LPS significantly increased adhesion and rolling after 5 h. LPS from *P. aeruginosa* took longer to increase adhesion when compared to LPS from *E. coli* and did not significantly increase rolling.	[47]

## 5. Intravital Imaging of the Lungs

### 5.1. Methods

LPS is commonly used to study the pathophysiology of lung inflammation, ALI, and the subsequent development of acute respiratory distress syndrome. However, depending on the route of administration, the mechanisms of pathophysiology differ. Laboratory animals challenged intravenously or intraperitoneally with LPS result in indirect lung injury through systemic inflammatory mediators [56]. Research targeting direct, unilateral, or bilateral injury of the lung has used i.n., intratracheal (i.t.), or inhalational models of LPS administration [56,57].

To successfully evaluate inflammatory changes in the lungs after the LPS challenge, intravital widefield fluorescence microscopy has adopted a vacuum-stabilized imaging system. This system consists of a coverslip mounted to a ring, forming an imaging window. Connecting the imaging window to a vacuum pump, the lung is visualized through wide-field microscopy following thoracotomy [58]. Applying this method, pulmonary damage and pathologies have been reported by various authors (Table 4).

### 5.2. Results

Wang et al. provide evidence regarding the differences in leukocyte recruitment in the pulmonary microvasculature between local and systemic endotoxic models [59]. Mice were either administered LPS intratracheally to mimic a local infection, or intravenously to mimic a systemic infection. It was reported that both models increased leukocyte accumulation and adhesion in the pulmonary microcirculation compared to PBS control. When comparing test groups, the systemic model found a greater accumulation of leukocytes than the local model. The two models found differing localization of trapped leukocytes: pulmonary venules for local inflammation and pulmonary capillaries for systemic inflammation. Additionally, the local model found a significant increase in the number of rolling leukocytes, while the systemic model did not [60]. Similar results were found in a model of intratracheal LPS-challenged hamsters where leukocyte rolling and adhesion were increased [60]. 1 h post-challenge, platelet sticking, and thrombi formation also occurred, and by 24 h leukocyte adhesion had significantly increased compared to the 1 h timepoint, and morphological changes were seen in endothelial cells.

Leukocyte rolling, adhesion, and functional capillary density (FCD) of pulmonary arteries and venules have also been investigated following inhalational administration of LPS [34]. Hall et al. reported no significant differences in leukocyte rolling or FCD in pulmonary venules and arteries between naïve and LPS-challenged mice. However, a significant increase in the amount of venular leukocyte adhesion was seen following the LPS challenge compared to naïve mice (Figure 1).

Parallel to these findings, Vasquez et al. examined the anti-inflammatory effects of doxofylline in LPS-challenged mice. They found an increase in leukocyte adhesion and rolling in the tracheal microcirculation following LPS administration. These results suggested greater cellular intravasation, which was confirmed by their histological data showing immune cell infiltration [61]. Additionally, the tracheal muscle yielded similar results, finding a significant increase in leukocyte sticking and rolling within the vessels.

Additionally, pulmonary microcirculation has been assessed for LPS-induced platelet aggregation by IVM. Cleary et al. found increased levels of platelet adhesion within the LPS inhalation group compared to the PBS control [62]. Pulmonary microcirculation findings, including increased platelet aggregation, neutrophil recruitment, and thrombi, were confirmed in mice 4 h after intraperitoneal injection of LPS with the additional assessment of neutrophil extracellular traps [63].

Lastly, blood vessel glycocalyx thickness and permeability, using an FITC-Dextran exclusion technique, have been assessed using IVM. Comparing aerosolized LPS and control mice after 8 h and 24 h, data from Margraf et al. suggests a decrease in pulmonary endothelial surface layer thickness, increasing vascular permeability [64].

**Table 4:** Impact of LPS administration on IVM readouts in the lung microcirculation.

Animal Strain	Dosage of LPS	Readout	Results	References
C57BL/6 mice	2 mg/kg, i.v. or i.t.	Leukocyte rolling and trapping	Local LPS significantly increased leukocyte rolling but only marginally increased after systemic LPS. Systemic LPS significantly increased leukocyte trapping in capillaries.	[59]
Hamsters	10 mg/kg, i.t.	Leukocyte rolling adhesion	LPS significantly increased leukocyte rolling and adhesion on the endothelium.	[60]
C57BL/6 mice	5 mg/kg, i.n.	Leukocyte adhesion, rolling and FCD	LPS significantly increased venular leukocyte adhesion. No significant change was seen in leukocyte rolling or FCD.	[34]
BALB/c mice	10 μg/mouse, i.n.	Leukocyte adhesion and rolling	LPS significantly increased leukocyte rolling and adhesion to the vascular wall.	[61]
BALB/c mice	5 mg/kg, i.n.	Platelet adhesion	LPS significantly increased platelet adhesion in the lung microvascualture,	[62]
C57BL/6	500 μg/mL LPS inhaled for 30 min at a flow rate of 15 mL/min	Glcocalyx width	LPS significantly decreased the width of vascular glycocalyx.	[64]

## 6. Limitations and Recommendations

Although IVM is a valuable tool for studying in vivo inflammatory processes in real-time, it is not without drawbacks. The preparation process required for IVM imaging is invasive. Animals must be anesthetized during the surgical procedure, and the administration of anesthetics can disrupt normal physiology, affecting respiration and hemodynamic conditions [65]. IVM requires surgically exposing the organ of interest, which can impact the overall physiology of the organ and the integrity of the surrounding tissue, leading to increased local and sometimes systemic inflammation [65]. Some organs, for example, the intestines, are exteriorized from the body to limit movement and improve image quality. Care must be taken to minimize the surgical procedures so that physiological conditions can be maintained as much as possible. For organs that cannot easily be exteriorized (i.e., the bladder), only a portion of the organ can be imaged. This limited field of view decreases the generalizability of the findings. Without a comprehensive view of the entire area, it is possible that the exposed portion of the organ is not representative of the entire organ.

Beyond surgical procedures, motion artefacts must also be minimized to ensure that clear images can be taken. This often involves pressure on the organ or surrounding tissue and the use of microstages or organ holders. These techniques limit the movements of the tissue caused by breathing or other physiological processes, which allows for better focus and clearer images. However, holding an organ or tissue in place alters the blood flow to that area, which can lead to hypoxia and even damage the tissue [66]. To accurately capture microcirculatory processes during IVM, a balance must be maintained between minimizing motion artefacts and maintaining normal physiological conditions.

Even with the organ exposed and immobilized, IVM has a limit to its depth of tissue penetration. Confocal microscopy can visualize tissues up to 80 μm until the tissue scatters light and a clear image is unable to be produced [67]. This limits the types of tissue that can be visualized using IVM, especially in larger animals. Even in smaller animals, this depth may not be enough to penetrate the entire organ, allowing only a portion of the organ to be visualized. For example, the skeletal system poses a unique challenge as light is absorbed, scattered, and dispersed by thick mineralized bone matrix and adipose-rich bone marrow. Imagining the brain requires invasive methods to thin the skull or implant imagining windows. Recently, Bhattacharyya et al. (2023) have developed methods for minimally invasive imaging of the murine tibia. In this protocol, a skin incision exposes the tibia, and image acquisition uses an imagining chamber constructed with thermoconductive T-putty. Imagining sessions can take place for up to 12 h and can then be repeated over multiple timepoints by closing the skin incision [68].

Although some studies use imaging windows for repeated observations, IVM is frequently a terminal procedure. Imaging is done once before euthanasia, or sometimes a few times within a small timeframe before euthanasia. It is therefore difficult to compare results over time, as repeat imaging of the same animal is not available. In addition, the IVM procedure causes added stress on the body, which can affect the physiology of the microcirculation. Heartbeat and circulation both slowdown, which affect microcirculatory processes. This is of concern, especially if the animal begins to die before the image-capturing process is complete. Since changes in the microcirculation are the main outcome used to compare experimental groups studied by IVM, if one animal begins dying sooner than others, differences in microcirculatory parameters could be observed that are not results caused by the experimental conditions.

## 7. Conclusions

Intravital fluorescence microscopy is a valuable tool in the realm of biological research, allowing for real-time observation of cellular and biological processes within live tissues. The ability to visualize alterations in leukocyte–endothelial interactions and perfusion across various live organ systems has allowed for insights into the characterization of immune responses. This review discusses the common methodology of IVM and synthesizes the collective knowledge surrounding LPS-induced inflammation in diverse organ models, including the brain, intestines, bladder, and lungs. Future research is required to minimize the limitations of this methodology and to increase the ability of IVM to continue to contribute to the study of inflammation.

## Data Availability

No new data were created or analyzed in this study. Data sharing is not applicable to this article.
